# Choriocarcinoma After Term Pregnancy With a Subsequent Successful Pregnancy: A Rare Entity

**DOI:** 10.7759/cureus.47583

**Published:** 2023-10-24

**Authors:** Ioakeim Sapantzoglou, Maria Giourga, Alexandros Psarris, George Daskalakis, Ekaterini Domali

**Affiliations:** 1 Obstetrics and Gynecology, ‘Alexandra Hospital’, University of Athens, Athens, GRC; 2 1st Department of Obstetrics and Gynecology, General Hospital of Athens ‘Alexandra Hospital’, National and Kapodistrian University of Athens, Athens, GRC; 3 Gynecology, Anti-Cancer Hospital of “Saint Savvas”, Athens, GRC; 4 1st Department of Obstetrics and Gynecology, National and Kapodistrian University of Athens, Athens, GRC

**Keywords:** choriocarcinoma, gtn, gestational trophoblastic neoplasia, gestational trophoblastic disease (gtd), gestational trophoblastic disease chemotherapy fertility choriocarcinoma mole

## Abstract

Gestational trophoblastic neoplasia (GTN) is a group of pregnancy-related disorders that arise from the cells of conception. They include gestational choriocarcinoma (CC), placental site trophoblastic tumor, and epithelioid trophoblastic tumor with these forms arising from a molar pregnancy, abortion, or a normal genetic pregnancy. Most cases of GTN are diagnosed when the serum hCG levels plateau or rise in patients being followed up after the diagnosis of hydatidiform mole but can also be suspected due to persistent vaginal bleeding after a normal pregnancy and delivery. Early diagnosis and treatment are pivotal for ensuring optimal outcomes and given the rarity of the disease, clinical management and treatment should be provided in specialized centers. Here, we present a rare case of a 31-year-old woman diagnosed with choriocarcinoma with pulmonary metastasis following an uncomplicated full-term pregnancy. After the suction evacuation and curettage, she underwent six cycles of chemotherapy with an excellent response, a fact that resulted in a subsequent pregnancy and birth without complications, occurring 18 months thereafter.

## Introduction

Gestational trophoblastic disease (GTD) entails a group of conditions spanning the pre-malignant diseases of complete and partial hydatidiform mole to the malignant entities of invasive mole, choriocarcinoma (CC), placental site trophoblastic tumor, and epithelioid trophoblastic tumor (ETT). The malignant forms of GTD are collectively called gestational trophoblastic neoplasia (GTN) [1].

The incidence of molar pregnancies is reported to be 0.2-1.5 per 1000 births in Europe [1]. CC appears to have an estimated overall incidence of one case per 50,000 pregnancies [2], which usually develops after molar pregnancies, with an incidence after full-term pregnancies reported by some to be as rare as one in 160000 [3].

This case report describes the unusual clinical presentation and successful management of a 31-year-old woman diagnosed with CC with pulmonary metastasis following an uncomplicated full-term pregnancy. After performing a suction evacuation and curettage procedure to remove the malignant tissues from the endometrial cavity, she underwent six cycles of EMA-CO (etoposide, methotrexate, dactinomycin, cyclophosphamide, vincristine) chemotherapy with an excellent response and an uncomplicated follow-up. As a result, the woman was able to conceive 1.5 years later and, after an unremarkable pregnancy, she delivered vaginally a healthy baby.

## Case presentation

A 31-year-old G1P1 female, one month after the delivery of a healthy newborn, presented to the emergency department of a tertiary hospital in Athens, Greece, with severe, acute-onset occipital headache and numbness of the left upper extremity. She underwent an emergency brain computerized tomography (CT) scan that revealed the presence of a subarachnoid hemorrhage and an arteriography demonstrated the presence of an aneurysm at the distal end of the frontal branch of the right middle cerebral artery, findings that were not associated with the prior pregnancy. She had a successful endovascular repair on the same day.

During her hospitalization, she complained of left-sided pleuritic chest pain before her admission to the hospital. Her overall clinical picture, along with increased levels of D-dimers (DD), led to performing a CT pulmonary angiogram, which did not confirm pulmonary embolism but revealed multiple nodules bilaterally (maximum diameter: 10 mm), as well as pleural effusion and atelectasis in the lower segment of the lungs bilaterally. Αn assessment was conducted with solid recommendations for further investigations as soon as she was to be discharged from the hospital.

One month later, she presented to the emergency department of the same hospital with fever, chills, and rigors. She was admitted to the hospital, and brain and chest CT scans were performed. The brain CT scan showed lysis of the ischemic findings and edema with no active bleeding. The chest CT scan revealed the remission of the pleural effusions but also an expansion and intensification of the known nodules. A whole blood and imaging work-up revealed high levels of beta-human chorionic gonadotropin (b-hCG; >225,000 IU/mL). Α transvaginal ultrasound scan showed increased uterus dimensions and the presence of a distended uterine cavity containing complex echogenic fluid, suggestive of the endometritis-pyometra complex. Abdominal and pelvic magnetic resonance imaging demonstrated a heterogeneous mass within the myometrium extending into the endometrial cavity measuring 8.5 x 7 cm. The lesion included a solid area in its periphery, cystic areas suggestive of high-velocity vessels, and a centrally positioned necrotic area. Thus, both the blood and imaging studies indicated a diagnosis of malignant trophoblastic neoplasia. During her hospital stay, the patient remained febrile (up to 39.5 °C) and developed respiratory distress, anemia, leukopenia, and thrombocytopenia. A U/S echocardiogram revealed pulmonary hypertension and a blood film assessment showed findings suggestive of microangiopathic hemolytic anemia. Due to the final diagnosis and the deterioration of the patient, she was transferred to our unit for further management and follow-up.

**Figure 1:**
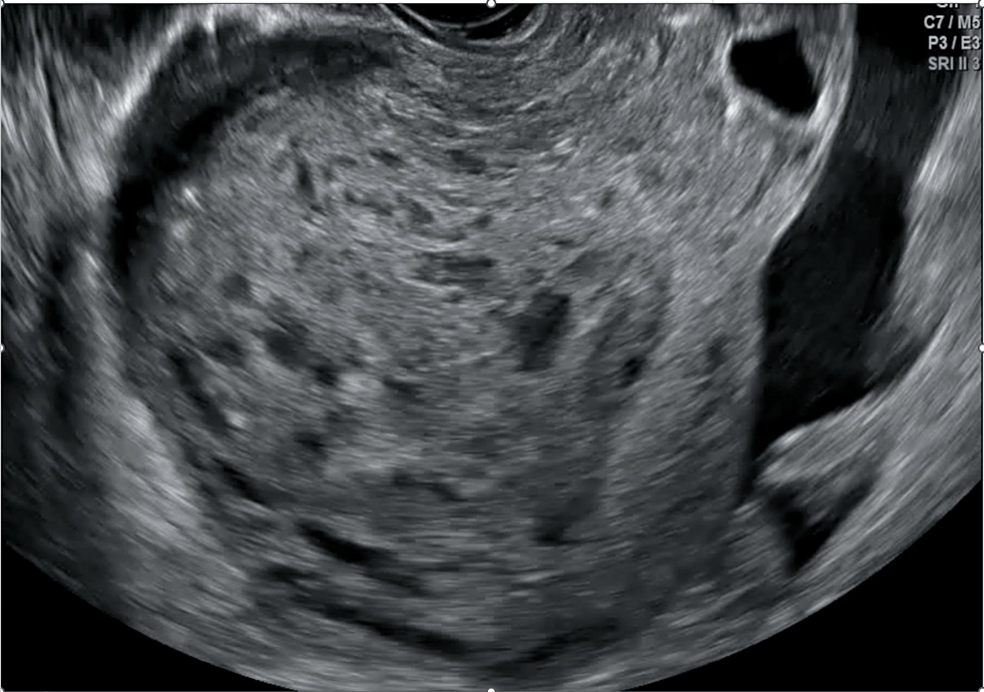
Transvaginal ultrasonography demonstrating an enlarged uterus with solid and cystic components within the endometrial cavity that penetrate the myometrium, indicating the presence of choriocarcinoma

**Figure 2:**
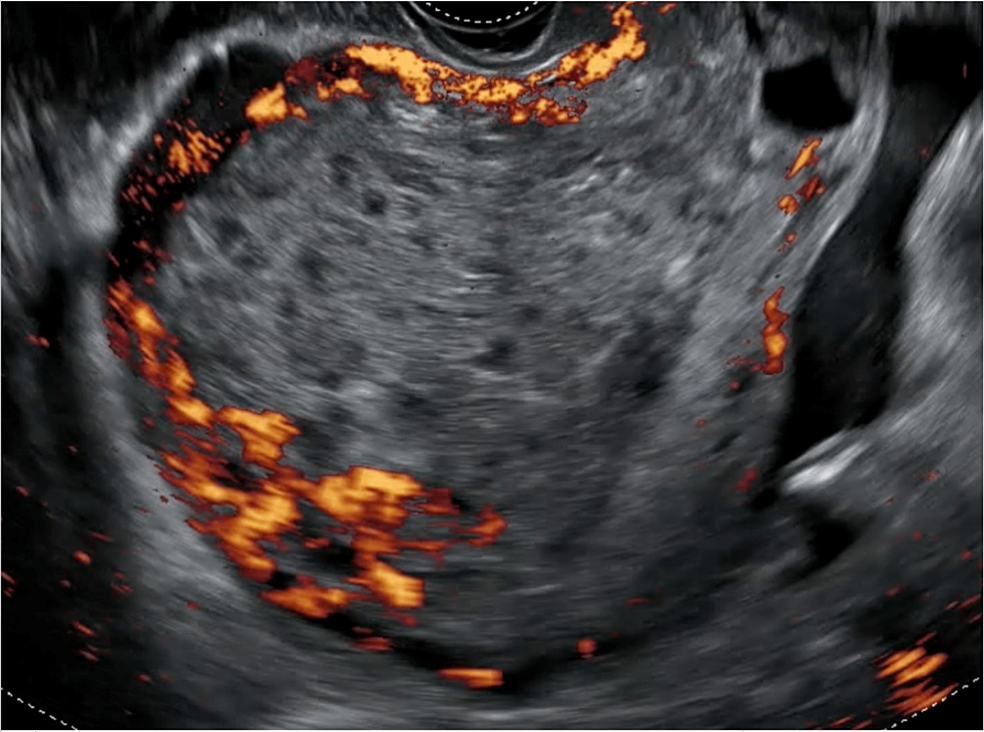
Color Doppler ultrasonography exhibits high vascularization of the endometrial lesion

In our department, after the initial workup and assessment (preoperation b-hCG: 232085 IU/mL), a transvaginal ultrasound was performed and a multidisciplinary team meeting (MDT) was held. The initial transvaginal ultrasonography revealed an enlarged uterus with solid and cystic highly vascularized content inside the endometrial cavity that invaded the myometrium (Figures 1, 2). The uterine evacuation process can be seen in Video 1.

**Video 1:** Transabdominal ultrasound video illustrating the process of uterine evacuation

Her International Federation of Gynecology and Obstetrics (FIGO) score was 8 (high risk) (Table 1). The patient was FIGO (International Federation of Gynecology and Obstetrics) stage IV.

**Table 1:** Patient's FIGO score b-hCG: beta-human chorionic gonadotropin; FIGO: Federation of Gynecology and Obstetrics

FIGO Criterion		Our patient’s score
Age (years)	<40 years old	0
Index pregnancy	normal	2
Interval from index pregnancy	<4 months	0
Pretreatment b-hCG	>10^4^	2
Largest tumour size including the uterus	>5 cm	2
Site of metastases	lung	0
Number of metastases identified	>8	2

Suction, evacuation, curettage, and multiple-agent chemotherapy were recommended; the patient was informed of these procedures and gave consent for them to be performed. After the suction evacuation and curettage were performed, a sample of her uterine contents was sent for histological analysis, which revealed, microscopically, large areas of hemorrhage and necrosis, as well as atypical mononuclear intermediate trophoblastic cells in sheets without any chorionic villi detected, which are morphological characteristics indicative of CC. After the procedure, her b-hCG levels showed a rapid decrease (postoperative day 1 b-hCG: 97521 IU/mL). The EMA-CO (i.e., etoposide, methotrexate, dactinomycin, cyclophosphamide, and vincristine) chemotherapy protocol was initiated on the second postoperative day. Two courses of induction chemotherapy with low-dose etoposide and cisplatin were initially given due to the significant risk of intracranial hemorrhage attributed to the recently operated brain aneurysm, followed by six cycles of the EMA-CO regimen, as per protocol, with complete remission of the disease. The patient's b-hCG levels were normalized after three cycles of the EMA-CO regimen and three additional courses were provided after that. The patient experienced no major adverse events other than alopecia and nausea and vomiting, which were managed with anti-emetics.

The patient was strongly advised to avoid pregnancy by using effective contraception (oral contraceptive pills, progesterone-only pills, implants) during her follow-up. However, the patient mentioned during her follow-up visits that her contraception consisted solely of condoms and withdrawal techniques. Her b-hCG levels during her follow-up period were the following: one week postop: 22 IU/mL; two weeks postop: 19 IU/mL; three weeks postop: 15 IU/mL; one month postop: 13 IU/mL; two months postop: 12 IU/mL; three months postop: 11 IU/mL; three months postop: 9 IU/mL; four months postop: <5 IU/mL; five months postop: <5 IU/mL; six months postop: <5 IU/mL. 

A whole-body positron emission tomography scan was performed with no atypical metabolic activity detected, after which follow-up cessation was decided.

Sixteen months after the end of her follow-up, the patient presented to our early pregnancy unit, being six weeks pregnant. An early pregnancy scan confirmed an intrauterine gestational sac and a fetal pole measuring 8 mm (crown-rump length: 8 mm) with regular cardiac activity. No signs of molar pregnancy were detected, and the woman had a regular antenatal assessment. The pregnancy was uneventful until the third trimester, in which a fetal ultrasound assessment revealed that the weight of the fetus was smaller than expected based on the appropriate values for the gestational age, i.e., estimated fetal weight at the 9th centile with normal Doppler parameters). Therefore, a tighter surveillance protocol was implemented.

She eventually had an uncomplicated vaginal delivery at 39+4 weeks of gestational age after induction of labor, as per our department's protocol, due to stagnant growth, and gave birth to a healthy female newborn. She had weekly b-hCG monitoring for three weeks with harmful levels and a monthly serial assessment without any abnormal increases. As of the current time, both the patient and her infant are in a state of good health.

## Discussion

CC after a full-term pregnancy is a rare entity [4], with its clinical suspicion being prompted mainly by increased b-hCG levels and postpartum hemorrhage. However, in some cases, the intuition is clinically established due to symptoms induced by metastatic lesions (e.g., headache, brain hemorrhage, abdominal pain, cough, hemoptysis, pleuritic chest pain, and respiratory distress). Final confirmation of the diagnosis is achieved after a histological examination of the placenta or endometrial curetting.

Among asymptomatic patients, CC is an incidental finding after examination of the placenta due to preceding pregnancy adversities (e.g., feto-maternal hemorrhage, stillbirth, and fetal growth restriction). In our case, as the pregnancy was completely uneventful, there was no clear indication for the histological examination of the placenta. However, as mentioned earlier, it should be highlighted that in cases of adverse events the macroscopic and, most importantly, microscopic examination of the placenta could provide valuable information for the establishment of the final diagnosis and the consultation of couples. Furthermore, contrary to the usual clinical appearance, our patient did not have any irregular bleeding, and she only showed signs and symptoms involving the respiratory tract due to the presence of lung metastases, a fact that delayed diagnosis and treatment. Therefore, as only 50% of GTN follows a molar pregnancy-25% may occur after spontaneous abortion or ectopic pregnancy while the rest may occur after preterm or term pregnancy-it should be considered in the differential diagnosis of patients with unusual clinical presentations, and hCG levels should be part of the formal investigation of such women.

The prognosis of CC is usually excellent, provided that some prerequisites are met. An early diagnosis should be established, appropriate referral to a specialized center should be made, and the proper chemotherapeutic regimen should be provided promptly [5]. According to the above, current evidence suggests that delays in diagnosis, such as those noticed in cases of CC after non-molar pregnancies, worsen the overall prognosis [6]. In 2000, FIGO introduced not only the criteria and tools for the diagnosis and investigation of GTN (Tables 1, 2) but also a scoring system (known as the FIGO score) to classify patients as low or high risk - a stratification that will determine the regimen that needs to be administered (Table 3) [7]. Furthermore, FIGO introduced a staging and classification system in 2009 that depends on the extension of the primary tumor and the presence and site of distant metastases (Table 4) [8]. Patients with a FIGO score of <6 are considered low-risk and require single-agent chemotherapy such as methotrexate. In contrast, patients with a FIGO score of >7 are treated with multiple-agent regimens, with EMA-CO being the most widely used regimen. This approach has led to overall remission rates of approximately 98%-100% [9]. Following the completion of chemotherapy in both high- and low-risk patients, patients should be monitored serially for 12 months.

**Table 2:** FIGO criteria for the diagnosis of post-molar gestational trophoblastic neoplasia hCG: human chorionic gonadotropin; FIGO: Federation of Gynecology and Obstetrics

FIGO criteria for the diagnosis of post-molar gestational trophoblastic neoplasia.
· Serum hCG plateau over 4 weekly measurements (day 1, 7, 14, and 21)
· More than 10% rise in serum hCG level of three consecutive weekly measurements (day 1, 7, 14)
· Elevated serum hCG 6 or more months after evacuation of the mole
· Histologic diagnosis of choriocarcinoma

**Table 3:** Tools for the investigation of gestational trophoblastic neoplasia

Tools for the investigation of gestational trophoblastic neoplasia
· Chest X-ray is appropriate to diagnose lung metastases and can be used for counting the number of lung metastases to evaluate the risk score
· Liver metastases may be diagnosed by ultrasound or CT scanning
· Brain metastases may be diagnosed by MRI or CT scanning

**Table 4:** International FIGO/WHO prognostic scoring index for gestational trophoblastic neoplasia b-hCG: beta-human chorionic gonadotropin; FIGO: Federation of Gynecology and Obstetrics

	International FIGO/WHO Prognostic Scoring Index for Gestational Trophoblastic Neoplasia		
Score	0	1	2
Age	<40y	³40y	
Antecedent pregnancy	Mole	Abortion	Term
Time since pregnancy	<4mo	4-6mo	7-12mo
Initial b-hCG levels	<1000	1000-9999	10000-99999
Largest tumor size (including uterus)	<3cm	3-4cm	5 or more cm
Size of metastases	Lung, vagina	Spleen, kidney	Gastrointestinal tract
Number of metastases	0	01-Apr	05-Aug
Prior failed chemotherapy	None		Single drug

Table 5 shows the FIGO anatomical staging of trophoblastic tumors. 

**Table 5:** FIGO anatomical staging of trophoblastic tumors FIGO: Federation of Gynecology and Obstetrics

Stage	Description
I	Gestational trophoblastic tumors strictly confined to the uterine corpus
II	Gestational trophoblastic tumors extending to the adnexa or to the vagina, but limited to the genital structures
III	Gestational trophoblastic tumors extending to the lungs, with or without genital tract involvement
IV	All other metastatic sites

Regarding the long-term outcomes of CC, the current literature is quite promising, with roughly 80% of women managing to achieve a further successful pregnancy following single- or multiple-agent treatment, as seen in our case [9]. However, menopause can occur earlier than expected for women treated with multiple-agent chemotherapy, with 13% and 36% of such patients experiencing premature menopause by the ages of 40 and 45 years., respectively. Although women should be informed of the slightly increased risk of stillbirth in subsequent pregnancies, they should be counseled that they can expect similar pregnancy outcomes compared to the general population [10].

Finally, health practitioners need to be aware that surveillance in future pregnancies can include pathologic analysis of the placenta for evidence of GTD and b-hCG level assessment at six weeks postpartum.

## Conclusions

CC is a rare condition, and its diagnosis requires a high level of suspicion by the clinician. The combination of obstetrical history, clinical symptoms and signs, biochemical assays, and ultrasound findings eventually establish its diagnosis, which should be prompt, for treatment to begin without delay. Clinicians should be aware of the atypical presentations of the condition such as its presence after an uncomplicated term pregnancy or the presence of symptoms from organs affected by the metastases of the malignant cells, the importance of FIGO staging for the management to be guided, and the need for treatment to be individualized. Appropriate management and the centralization of care for such an uncommon disease are essential, as optimal and timely treatment, which consists of the correct diagnosis and staging, appropriate chemotherapy regimen, and proper follow-up, has proven to improve these women’s cure rates and subsequent pregnancy success rates.
